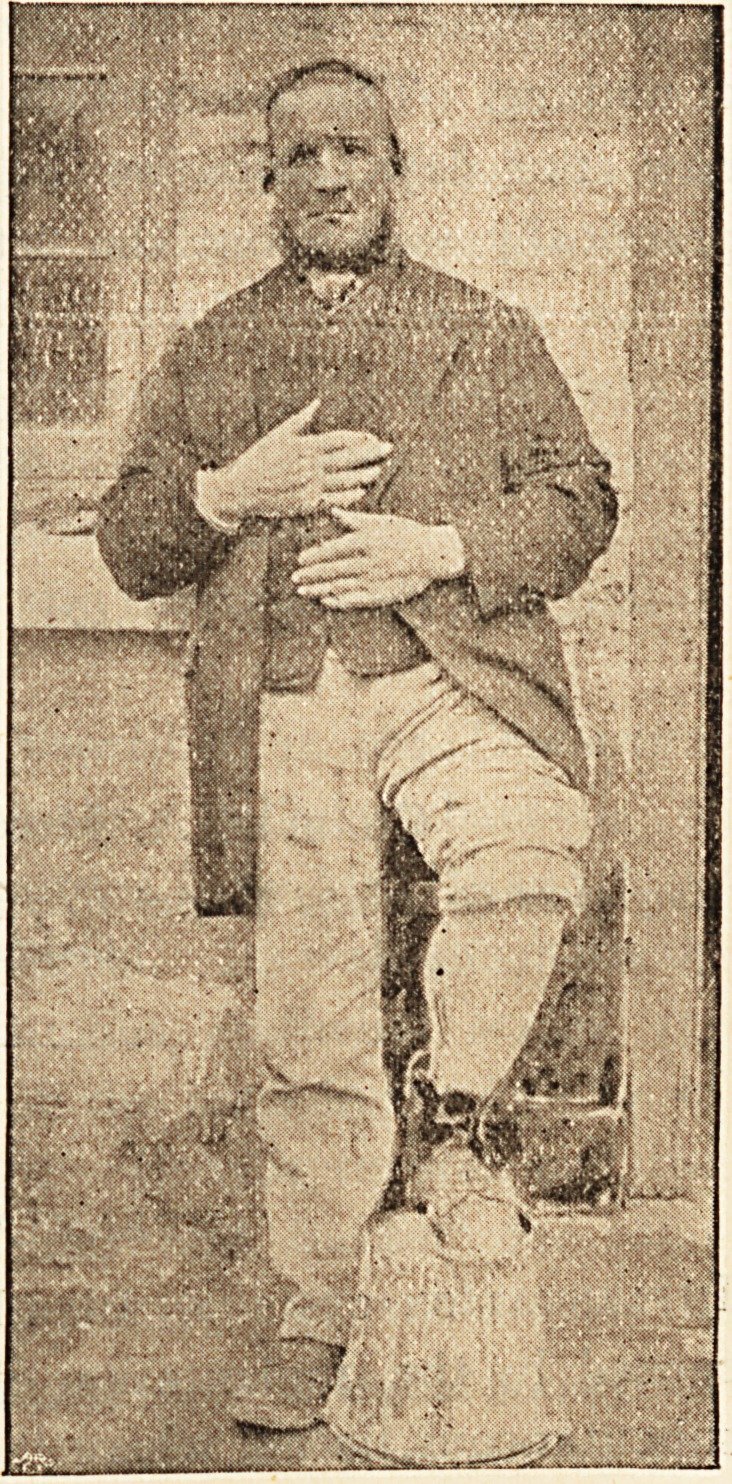# Case of Myxœdema

**Published:** 1894-09

**Authors:** W. A. Hayes


### CASE OF MYXŒDEMA.

BY

W. A. HAYES, M.R.C.S. Eng., L.R.C.P. Lond.

---

E.S., aged 47. For a very long time the patient was considered to be suffering from chronic Bright's disease, chronic rheumatism, and a variety of other complaints.

Early in 1894 I examined the patient, and found him to be suffering from myxœdema. He complained of weakness, was troubled with indigestion, pains in his limbs, sweating confined to the head; he could not grasp his tools when working, and he could do very little work as he got so tired. His speech was very slow and deliberate: if asked a question, he would be a long time beginning to answer it. The hair on his head and face was badly nourished and scanty. The right heart was dilated. He felt cold, and easily caught cold, on one occasion having bronchitis. He weighed 14 stone 1 lb. Measurement round calf of leg, 19¼ inches. Urine: specific gravity 1030, no sugar, no albumen. Lithates and phosphates present.

*Feb. 19th.*—Glycerine extract of thyroid was begun in doses of two drachms thrice daily after food.

*Feb. 20th.*—Patient "felt his head bad," and "cold sensations about his body."

*Feb. 25th.*—Pain in head less. Complains of a "feeling of cold water running under his skin." Feels his skin softer and more comfortable. His food "goes better," and his "stomach is more comfortable."

*March 3rd.*—He can bend his fingers and grasp better: his skin generally "feels looser." As yet no improvement in locomotion, appearance, or speech. The calves of the legs, which on Feb. 19th were very hard, are a little softer. The skin is cracking over back of hands and arms.

*March 6th.*—Desquamation almost general. On the backs of hands it is very well marked, large flakes coming away. Patient feels very much better. Finds the glycerine extract gives him headache if taken as directed, so only took it twice daily for the last two days.

*March 12th.*—He has had no extract for three days. To-day he began taking three of Burroughs and Wellcome's tabloids, each of which is "equal to five grains by weight of healthy thyroid gland."

Patient's legs are much softer, he can walk better, does not get tired so quickly as he did a month ago. Looks better.

At this time I was unable to see him again. The gentleman doing my work found three tabloids produced a temperature of 103.2°, violent headache, anorexia, and a feeling of sickness; he stopped them for a day or two, and began again with one twice daily. This treatment he continued. The patient about the middle of April began to do a little work, which tired him very much at first.

*May 1st.*—I again saw the patient. He weighs 11 stone 3 lbs.—a loss of 40 lbs. in 70 days. He is practically well. He has a thick growth of hair on his head. The measurement round calves is 16½ inches. He can speak quickly and converse freely. Complains of nothing but feeling a little weak, and that not so much as a month ago. He has a good, fresh colour, a soft and natural skin, and an appearance so altered that his friends who have not seen him for some time hardly recognise him. He can work well.

*May 19th.*—Patient still improving in strength. Ordered two tabloids a week.

*June 8th.*—Patient is in almost constant work, and complains of nothing. The two tabloids a week seem to be enough. It is now 110 days since he began thyroid feeding. He was practically well about the 60th or 70th day.

## Figures and Tables

**Figure f1:**
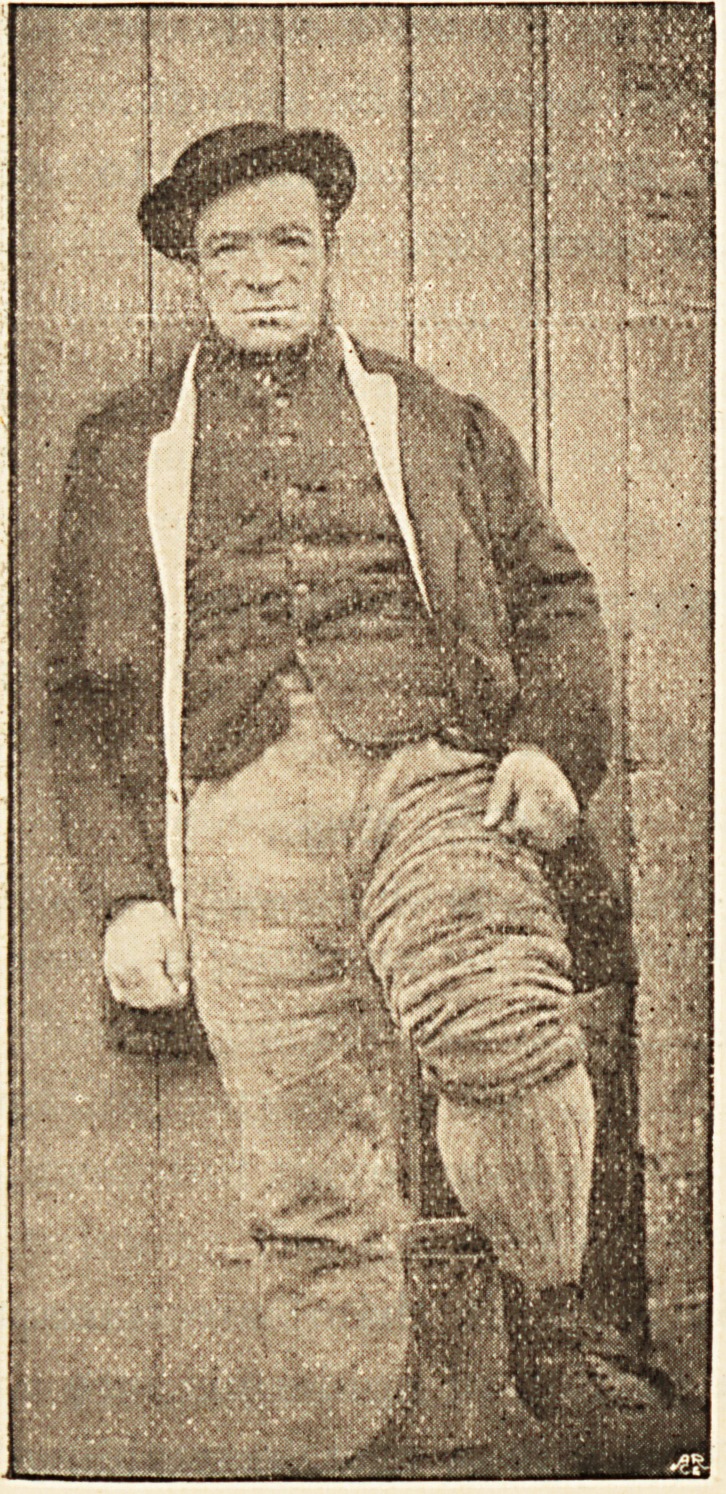


**Figure f2:**